# Soft Robots for Ocean Exploration and Offshore Operations: A Perspective

**DOI:** 10.1089/soro.2020.0011

**Published:** 2021-12-06

**Authors:** Simona Aracri, Francesco Giorgio-Serchi, Giuseppe Suaria, Mohammed E. Sayed, Markus P. Nemitz, Stephen Mahon, Adam A. Stokes

**Affiliations:** ^1^ Scottish Microelectronics Centre, Institute for Integrated Micro and Nano Systems, School of Engineering, The University of Edinburgh, Edinburgh, United Kingdom.; ^2^ Institute of Marine Sciences—National Research Council (ISMAR-CNR), La Spezia, Italy.; ^3^ Department of Chemistry and Chemical Biology, Harvard University, Cambridge, Massachusetts, USA.; ^4^ Robotics Engineering Program, Department of Mechanical Engineering, Worcester Polytechnic Institute, Worcester, Massachusetts, USA.

**Keywords:** ocean exploration, offshore operation, sustainable development, abyssal exploration, evolution of soft robots, oceanography

## Abstract

The ocean and human activities related to the sea are under increasing pressure due to climate change, widespread pollution, and growth of the offshore energy sector. Data, in under-sampled regions of the ocean and in the offshore patches where the industrial expansion is taking place, are fundamental to manage successfully a sustainable development and to mitigate climate change. Existing technology cannot cope with the vast and harsh environments that need monitoring and sampling the most. The limiting factors are, among others, the spatial scales of the physical domain, the high pressure, and the strong hydrodynamic perturbations, which require vehicles with a combination of persistent autonomy, augmented efficiency, extreme robustness, and advanced control. In light of the most recent developments in soft robotics technologies, we propose that the use of soft robots may aid in addressing the challenges posed by abyssal and wave-dominated environments. Nevertheless, soft robots also allow for fast and low-cost manufacturing, presenting a new potential problem: marine pollution from ubiquitous soft sampling devices. In this study, the technological and scientific gaps are widely discussed, as they represent the driving factors for the development of soft robotics. Offshore industry supports increasing energy demand and the employment of robots on marine assets is growing. Such expansion needs to be sustained by the knowledge of the oceanic environment, where large remote areas are yet to be explored and adequately sampled. We offer our perspective on the development of sustainable soft systems, indicating the characteristics of the existing soft robots that promote underwater maneuverability, locomotion, and sampling. This perspective encourages an interdisciplinary approach to the design of aquatic soft robots and invites a discussion about the industrial and oceanographic needs that call for their application.

## Introduction: Challenges for Aquatic Soft Robots in the Ocean Observing System and Offshore Monitoring

Scientists established the unfolding of climate change ∼40 years ago. Since then it has become increasingly important to understand the factors that influence the climate of our planet. The oceans play a key role in climate regulation: the sea is capable of storing energy and chemicals (e.g., CO_2_, O) and sustaining coastal populations and offshore human activities. In 2017, a United Nations factsheet estimated that 2.4 billion people live within 100 km of the coast.^1^ Nevertheless, we know more about the moon surface than the ocean floor.^2^ The best known water masses lie in the first 2 km beneath the ocean surface.

**FIG. 1.**
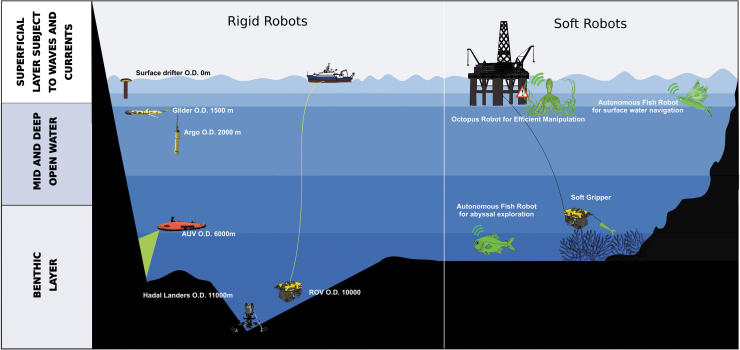
Distribution of the technology used presently to explore the ocean. Rigid robots are, at present, the majority of the devices, used for marine applications. This figure highlights potential applications where soft robots need to be used, which would be impossible/hard to achieve with rigid robots. The autonomous devices, for example, AUVs, gliders, drifters, and Argo floats, have limited battery life (up to 180 days). In addition to this Argo floats, drifters, and gliders are limited to the surface or the first 2 km of the water column. Only few AUVs in the world can reach 6000 m depth, and they have to follow a predetermined path. ROVs commonly used for seabed exploration and industrial surveys are tethered and need to be constantly remotely operated. Long-distance deep exploration is one of the limitations of currently available technology, where soft robots can come into play. The *right panel* depicts the present (grippers for coral reef sampling) and future applications of soft robots, all represented in *green*, as we envisage them. These applications encompass benthic exploration (e.g., autonomous fish robot), surface perturbed water navigation, and near-surface repair operations for offshore platforms. AUV, Autonomous Underwater Vehicle; ROVs, Remotely Operated Vehicles. Color images are available online.

Industry currently faces the challenge of making offshore sites safer, both for personnel and for the environment.^3,4^ This improvement in safety entails monitoring aging infrastructures to minimize the hazards associated with structural decay and human error.^3,5^ However, the harsh climate of the offshore environment renders working safely in these areas extremely daunting for human operators and conventional autonomous systems alike (Fig. 1). Therefore, industry is calling for the development of efficient solutions that offer increased autonomy and data collection capabilities,^6^ to sustain the thriving offshore renewable industry, whose assets are located in remote and high energy sites to facilitate the power takeoff.

**FIG. 2.**
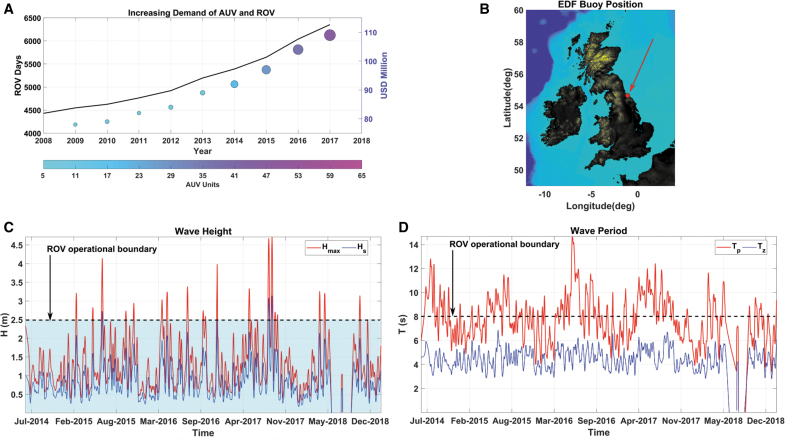
Industrial data show the increasing demand of offshore robots and the harsh conditions of an offshore site close to the United Kingdom coast. Image in **(A)** is inspired by Loffman^7^ (data from Westwood Douglas). The *circles* show AUV demand analysis; *color* and *size* of the *dots* represent the increase in AUV units demand in the oil and gas sector. The plot also shows an increase in ROV days (*black left axis*) and investments in US dollars (*violet right axis*). **(B)** Shows the location of an industrial moored buoy. The mooring is equipped to collect wave data **(C, D)**. The location of the buoy is close to the coast, yet the wave data indicate challenging weather conditions. The Douglas Sea Scale defines rough sea states as those with 2.5 m wave height. The *cyan area* indicates the 2.5 m threshold, exceeded 10% of the times. The operational boundaries for ROVs deployment are 2.5 m wave height and 8 s wave period, depending on location and vessel.^124^ The *dashed line* in (**D)** highlights the 8 s wave period threshold. Data courtesy: EDF Color images are available online.

Data (e.g., corrosion trends, structural vibration, algal cover, soil stability, and so on) are vital to assess the integrity of structures, to build accurate prognostic models, and to minimize human interventions. As a consequence, the use of robots, gathering data, during inspection, operation, and maintenance of offshore infrastructures, is growing (Fig. 2A).^7^

It is crucial to collect more data to investigate also the ecological and physical effects of anthropogenic structures in high seas. For instance, it is not clear yet how unused offshore assets could affect species migration, larvae spreading, and whether such structures would coherently interact with existent Marine Protected Areas.^5,8,9^ The offshore industry is increasingly involved in operations in deeper waters, where delicate ecosystems, such as cold-water coral reefs,^8,9^ hydrothermal vents, and sponge grounds,^10^ are subject to threat. Similarly, coastal waters, with their intricate and diverse ecological networks, are progressively exposed to increasing danger by human exploitation.

The expansion of human activities at sea has not been followed by a proportional development of data collecting subsea technology. Autonomous Underwater Vehicles (AUVs)—gliders—are ideal for long-distance travel at mid-depth—up to 1500 m—and Remotely Operated underwater Vehicles (ROVs) are designed for low-speed maneuvering far from the disturbance of the sea surface. Hence, despite the growth in the employment of underwater robots, technological constraints prevent their regular usage in the extreme oceanic environment, such as the two ends of the water column: in deep waters and in superficial, highly perturbed waters.

Within the scope of this article, we consider deep water the zones of the water column below 2000 m and/or the benthic layer. Current technological solutions prevent long-distance travelling close to the sea bottom and accurate slow-speed operations close to the surface.

This study considers these two frames of operation as exemplar case scenarios, where the employment of underwater soft robots could become a viable solution.^11–13^ In this perspective we convey the state of the art of aquatic soft robots^13–23^ suggesting that future soft robotic systems may provide a complementary approach to the use of standard robotics, addressing the challenges posed by abyssal exploration and automation of offshore systems.

## Applications

### Abyssal exploration and sampling

Most of the sea floor has been mapped to a 5 km resolution, which is sufficient to detect a large-scale submerged ridge, but is not enough to identify smaller-scale geological features, a ship, or plane wreck.^24^ The need to understand the role of the deep sea in the circulation^25^ and energy balance of the ocean^26^ is a pressing argument pushing for further abyssal exploration. Deep sea data will unveil a better knowledge of the deep marine environment and its urgency to be protected or its potential for a sustainable use.^27^

**FIG. 3.**
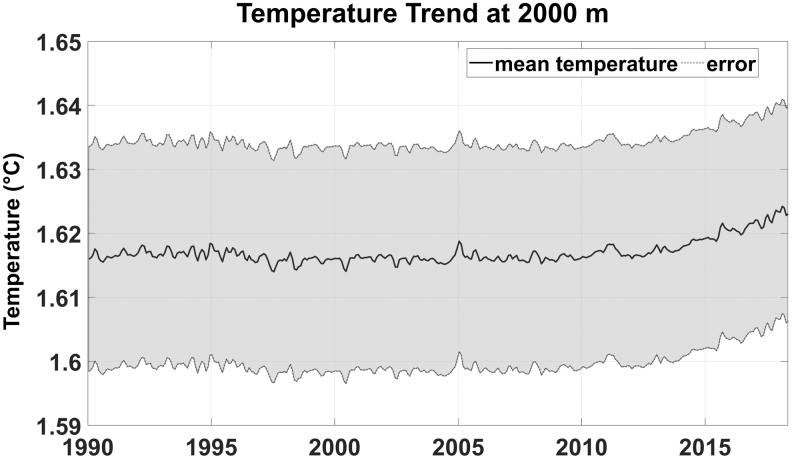
Increasing temperature trend at 2000 m depth. Data are from Global Ocean-Gridded objective analysis fields of temperature and salinity, using profiles from the reprocessed *in situ* global product CORA, using the ISAS software. Objective analysis is based on a statistical estimation method that allows presenting a synthesis and a validation of the dataset, providing a validation source for operational models, observing seasonal cycle, and interannual variability.

For instance, the uncertainty related to deep ocean temperature data (Fig. 3) could be reduced by increasing the amount of abyssal measurements.^28^ In addition, the leading dissipative terms that regulate the overall dynamical balance of the ocean still remain an open question in the understanding of the ocean general circulation.^29^ Momentum sinks are commonly associated with prominent features of the bottom topography, but smaller-scale elements of the order of tens of kilometers are being regarded as essential to achieve the full understanding of the driving forces of the climate.^30^

**FIG. 4.**
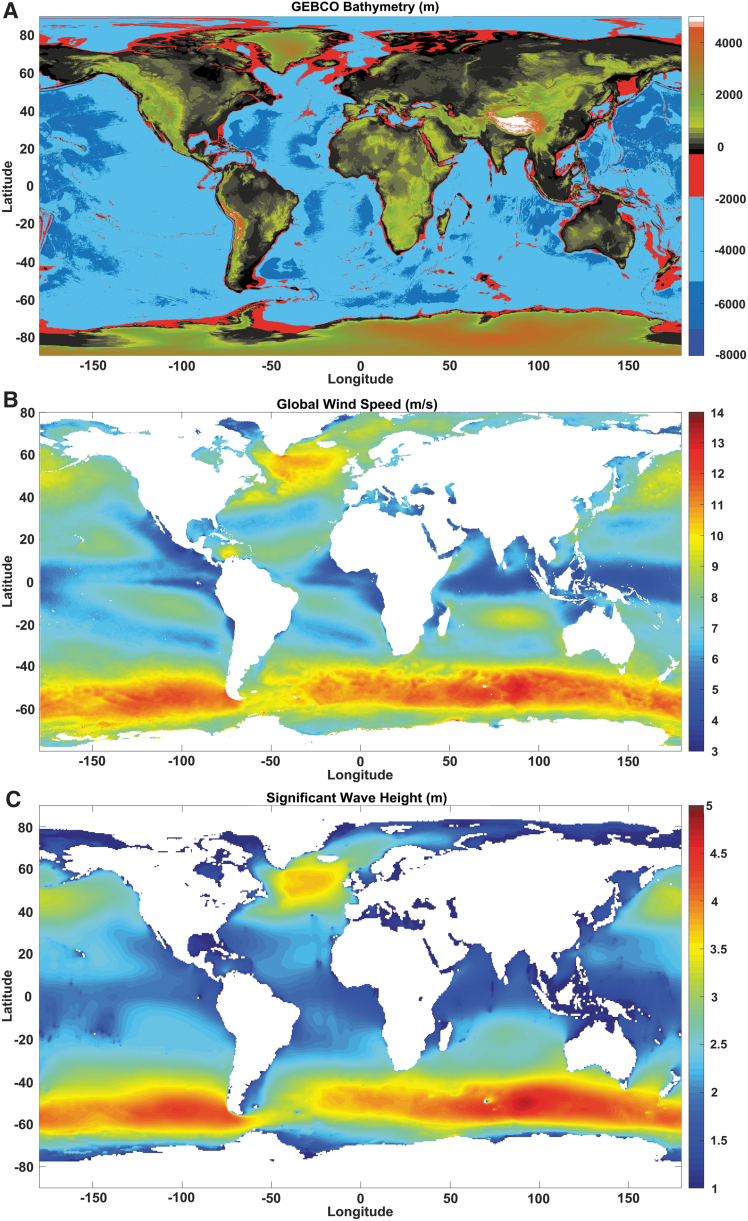
**(A)** Shows the General Bathymetric Chart of the Oceans bathymetry. Regions of the ocean shallower than 2000 m are highlighted in *red*. *Light blue color* shows ocean depths between 2000 and 5000 m, *blue* between 5000 and 6000 m, *indigo* indicates depth >6000 m. **(B)** Represents the global wind speed distribution. Data are from 2018. The climatology includes monthly averaged wind variables calculated over the global oceans. The gridded daily wind and wind stress fields have been estimated over global oceans from Metop/Advanced SCATterometer retrievals using the objective method. **(C)** Significant height of combined wind waves and swell from ERA5, the fifth generation of ECMWF reanalysis. Data are from 2018. Color images are available online.

However, these features are systematically unresolved by conventional global topographic datasets and by the general circulation models regularly used to investigate ocean dynamics. The availability of oceanographic data is too sparse to adequately characterize topographic terms, whose definition requires high-resolution flow measurements in remote and topographically complex areas.^31,32^ The lack of suitable ocean observing systems, to survey extended regions of the bottom layer of the ocean, Figure 4A, hinders the complete understanding of the ocean dynamics.

Existing AUVs are not able to perform long-range operations at very close proximity to the bottom of the ocean, thus highlighting the need for disruptive new technologies suited for persistent navigation adjacent to the bottom of the sea.

In observational natural sciences, such as bio-geophysical-oceanography, AUVs are often used to survey regions beneath the polar ice sheets to map large morphological features on the sea floor and to explore deep sea hydrothermal vents. Some of these operations have to be undertaken as close to the sea bottom as possible.^33^ When the bathymetry is not known well enough to allow the operator to program the AUV for a safe mission, or the basin is swept by strong currents, or the survey needs real-time data or physical samples, AUVs are not suitable. An analysis of the risks of AUV operations is reported in Brito *et al.*^34^ When physical samples or real-time data are necessary, explorers turn to ROVs, which are tethered and not autonomous and can only operate within a very limited range.

**FIG. 5.**
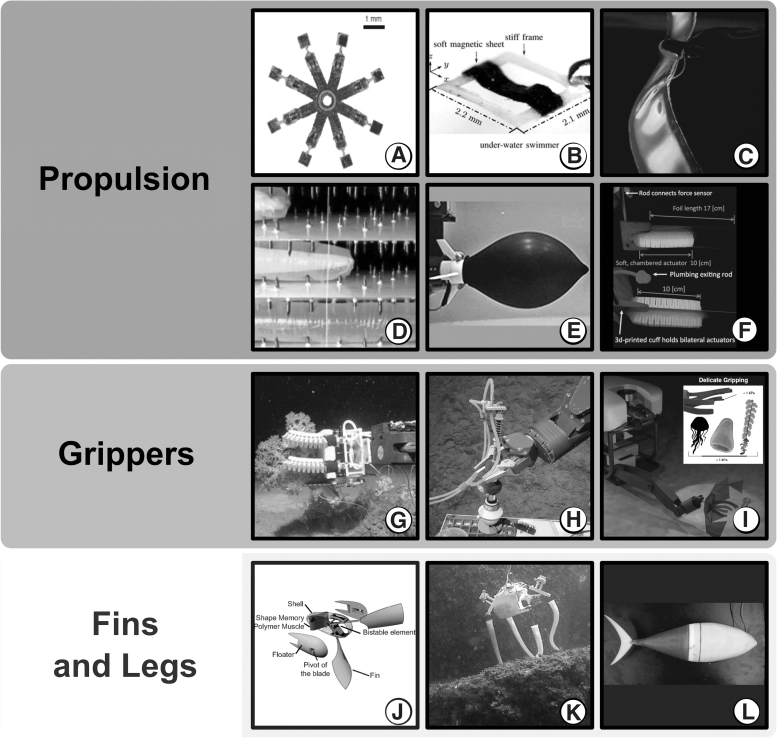
Some examples of underwater soft robots, which represent some of the aspects that could aid in ocean operations. In particular soft robotics advances brought to light the advantage of innovative propulsion methods allowed by soft materials, in the “Propulsion” panel. Few traditional robots exploited the capabilities of soft materials for delicate sampling, in the “Grippers” column. Soft materials can be moulded into fins and legs, which can stabilise the movement of a robot (soft or rigid) or allow locomotion, in the “Fins and Legs” section. **(A)** Multi-functional soft-bodied jellyfish-like swimming (Ren *et al.*^36^). **(B)** Untethered Miniature Soft Robots: Modeling and Design of a Millimeter-Scale Swimming Magnetic Sheet (Jiachen Zhang and Eric Diller^37^). **(C)** Translucent soft robots driven by frameless fluid electrode dielectric elastomer actuators (Christianson *et al.*^41^). **(D)** A soft robot that navigates its environment through growth (Hawkes *et al.*^42^). **(E)** Ultra-fast escape maneuver of an octopus-inspired robot (Weymouth *et al.*,^43^ image credit SMMI, University of Southampton.^40^). **(F)** Undulatory Swimming Performance and Body Stiffness Modulation in a Soft Robotic Fish-Inspired Physical Model (Jusufi *et al.*^44^). **(G)** Soft Robotic Grippers for Biological Sampling on Deep Reefs (Galloway *et al.*^45^). **(H)** Stronger at Depth: Jamming Grippers as Deep Sea Sampling Tools (Licht *et al.*^46^). **(I)** Ultragentle manipulation of delicate structures using a soft robotic gripper (Sinatra *et al.*^47^). **(J)** Harnessing bistability for directional propulsion of soft, untethered robots (Chen *et al.*^48^). **(K)** Hybrid parameter identification of a multi-modal underwater soft robot (Giorgio-Serchi *et al.*,^38^ photo credits Massimo Brega). **(L)** Tuna robotics: A high-frequency experimental platform exploring the performance space of swimming fishes (Zhu *et al.*,^39^ figure credits Christopher Tyree, University of Virginia School of Engineering).

The development of soft robotics technologies^35^ represents a unique opportunity to address these challenges, offering new perspectives in navigation, manipulation, propulsion (Fig. 5),^36–48^ and sensing. Soft materials: incompressible,^49^ resistant, compliant, and versatile^50^ can alleviate the risk associated with explorative missions of traditional robots.

Flanking traditional rigid robots with soft devices could aid underwater existing technology to accomplish hazardous tasks in the yet unknown oceanic environment.

The use of soft materials to constitute or protect core electronics could reduce the chance of a collision with unknown bottom or floating features to result fatal for an underwater mission. In contrast, relaxing security measures to avoid collisions would enable the collection of remote data in areas that fall out of the action map of completely rigid robots. Nevertheless, conducting this study, we did not come across with entirely soft robots capable to perform complex tasks in the environments that we identified as crucial for industrial sustainable development and oceanographic exploration.^51–54^ From our investigation emerged an increasing trend in embedding soft elements into established underwater technologies^45,46,55^ (Fig. 5G–I).

Furthermore, the inherent dexterity of soft materials empowered bioinspired propulsion, paving the way for novel navigation techniques achievable for soft robots^17,18,36,38,43,56–74^ (Fig. 5A–F, K). Even in case the propulsion would not entirely rely on the elongated body theory of fish locomotion,^17,39,47,57,61,62,75–87^ soft fins^21,22,79,88^ (Fig. 5J, L) and bladders^55^ can aid stabilizing the robots' navigation route and depth. As far as the composition of the used soft materials is concerned, recent progresses support the use of highly biodegradable blends,^89–98^ which would attenuate the environmental impact of those soft parts that will go lost or replaced.

At high depth, the impracticality of accurate manipulation control gives way to soft grippers (Fig. 5G–I), which can better deal with a larger variety of objects to be grasped and can account for fragile samples of complex shape. Coral reefs, for example, are one of the most delicate and important ecosystems of the planets^99,100^ and constitute a proxy for ocean acidification and warming. Hence, enabling autonomous sampling and monitoring of coral reefs is important for their safeguard. Navigation in the vicinity of a coral colony and handling of corals are extremely complex tasks, where soft grippers^45–47,101^ (Fig. 5G–I) and soft eversion robots^42,102^ prompt the advantage of a compliant mechatronic system.^42^

While robotics prototypes are progressively getting closer to their biological counterparts,^22,58,60,61,88,103–106^ these remain for the most part laboratory-scale experiments. Hence, if on one hand there is evidence that bioinspired soft robots are not simply an academic exercise, rather offering a clear advantage in terms of performance, on the other hand a major effort is still needed to drive the transition of these systems from prototypes to actual vehicles fit for operation at sea.

The employment of soft autonomous platforms roaming the depth of the oceans to perform high-resolution observation of the abyssal environment relies on new advanced sensing technologies fit for embedding in compliant structures.

In recent times, the interest in wearable devices has fostered the development of new flexible sensors and bioinspired technologies have further promoted the study of sensing technologies.

Recent examples entail whisker-inspired sensors,^107–113^ devices which replicate the flow diagnostic capabilities of the lateral line of fish^44,114,115^ and sensor-embedded wearable skin^116,117^ for marine mammals. These sensors are designed to be distributed as dense arrays over the surface of a body travelling underwater, thus enabling a better spatial description of the parameters of interest, as well as accurate prognostic of the state of the robots, for the purpose of control and localization. Given the importance that turbulence measurements of microstructure hold in the understanding of the nature of energy dissipation in the ocean,^118^ these new breed of sensors may unveil an unprecedented degree of information.

### Operations in highly perturbed surface waters

The surface of the ocean is a challenging environment, where standard operations (i.e., inspection and manipulation) become extremely impractical and dangerous due to the disturbances from waves and currents. Furthermore, offshore infrastructures are often located in environments subject to extreme weather conditions, making operation and maintenance of these systems especially costly and unsafe.

The World Meteorological Organization (WMO), adopting the Douglas Sea Scale,^119^ states that rough sea conditions are characterized by waves with heights of at least 2.5 m. The Beaufort scale considers winds above ∼10 m/s as a strong breeze.^120,121^ Figure 4B^122^ and C^123^ therefore shows the extension of the harshest regions on our planet (2018 data).

Wave data collected on the eastern coast of the United Kingdom (Fig. 2B–D) show how the coastal environments too pose considerable challenges to offshore infrastructures.

With the expansion in the ocean of human activities^124^ toward less accessible and more fragile environments, state-of-the-art underwater robotics technologies have progressively become less suited at coping with the increased degree of complexity of their missions.

As an example, commercially available robots are not suited for acquiring field measurements in the vicinity of submerged structures and performing even basic manipulation tasks when subject to stochastic currents and wave perturbations.^125^ A technological answer to this challenge, however, is still to be found. Existing vehicles are not suited for operating at low operational depth, where the effect of waves^126,127^ is so prominent that standard control techniques are not able to compensate for wave disturbances in order for autonomous systems to safely perform station keeping for inspection or manipulation.^128,129^ Similar problems arise in the case of vehicles operating close to submerged structure under the effect of superficial currents^130,131^ of <2 m/s.

In the rough offshore environment, the inherent structural flexibility of the robot will play an invaluable role in enabling a safe physical interaction^132,133^ with the submerged structures upon which the vehicle is operating. At the same time, compliance of the vehicle body will alleviate the computational burden and power otherwise required from the control and thrusters to maintain a safe distance. Evidence that these strategies represent viable solutions is the extensive work performed on compliant, tendon-driven manipulators capable of performing robust and firm grasp with minimal actuation^134–137^ and by recent work on advanced adhesion technologies.^138–140^

The performances of reversible adhesive systems suitable for operating on wetted and irregular surfaces are improving remarkably, and as suction forces in the order of 300 kPa become achievable,^141^ the chance to use these technologies to enable soft robots to work in the wave slamming region of an offshore platform becomes a reality.

The swimming skills of fish and cephalopods have inspired many of the advanced functional capabilities now encountered in soft robots. Enhanced stability during underwater legged locomotion^59,134–137^ and thrust augmentation due to added-mass variation^72,142,143^ are only two examples in the design of new soft unmanned underwater vehicles.

Thanks to emerging technology^144,145^ offshore assets can be autonomously, safely, and closely monitored.

The advantage of using soft robots for industrial applications lies in the fact that soft robots are low cost and easy to manufacture; they can navigate into restricted spaces where a hard robot would struggle. Soft robots can overcome the difficulties of such environment thanks to their compliant intrinsic nature.

## Discussion

The new generation of marine robots is not exempt from challenges. Some important issues that have to be addressed are energy consumption, autonomy, efficiency, sensing capabilities, memory, and pollution from polymers. Ubiquitous plastic pollution, ocean acidification, and chemical contamination are already heavily affecting ocean wildlife and coastal communities. Therefore, it is imperative to plan the future of marine soft robots minimizing their impact on the ocean.

Currently, soft robots do not meet the requirements for performing data collection in the remotest areas of the oceanic environment (Fig. 1).

Untethered underwater soft robots are rare; those controlled without the aid of tethers have an autonomy of only few tens of meters or less. Autonomy also includes power supply.

Existing marine devices (e.g., SBE 911plus CTD,^146^ SBE 19plus V2 SeaCAT Profiler CTD memory^147^) can live underwater for 6 months to 2 years, sampling at 24/4 Hz up to 10,500 m (tethered).

### Energy consumption and efficiency

The Energy consumption and efficiency of soft robotic systems have not been studied extensively in the literature.^148^ The ratio of the task-oriented output energy from the robot to the total energy input is known as the efficiency of the robot, and it is a key figure of merit for all machines.^148^ The energy input to soft robots is usually sourced from batteries, pressurized gas or liquid, or chemicals, and it is converted into useful work by the robot to actuate, locomote, crawl, climb, grasp, pick-up objects, jump, or sense.^148^ Energy efficiency is, thus, an important indicator for guiding the design and optimization of enhanced soft robotic systems.^149^ The energy efficiency can influence the choice of actuator, energy source, materials, structural properties, and locomotion mode and ultimately justify the use of soft mechatronics systems rather than a conventional machine.^149^

Soft robots are complex hybrids of chemical, pneumatic, hydraulic, mechanical, and electrical components and this complexity makes analyzing the efficiency and characterizing the energy losses in the system a difficult task.^148^

Most soft robots currently sit at a prototype level of development, making the assessment of their actual efficiency partly speculative.

According to an analysis of energy efficiency of mobile soft robots,^149^ the efficiency of most mobile soft robots in literature is low, with most robots having an efficiency lower than 0.1%.^149^ Nemiroski *et al.*^150^ reported an efficiency of 1–2% for a single joint of their soft robot “Arthrobot,” where the joint was an inflation-based elastomeric actuator. The remaining input energy to the system went into reversible expansion of the elastomer and irreversible losses.^150^

In general, inflation-based elastomeric actuators for soft robots have a low efficiency,^151,152^ and they are not only affected by the reversible expansion of the elastomer but also are influenced by the strain, strain rate, and viscous losses in the flowing gas.^148^ Another class of soft actuators—Vacuum-Actuated Muscle-inspired Pneumatic structures—which use deflation rather than inflation and operate at low strain levels, achieves a relatively higher efficiency of ∼27%.^153^ This efficiency value is comparable to human muscle efficiency (∼40%).^154^

Pneumatic soft actuators, particularly “Pneunets,”^14^ are very popular among soft robotic researchers for many different applications, despite their low efficiency.^155^ Analysis of PneuNet actuators with various wall thicknesses and different soft materials shows that the efficiency of these soft actuators lies in the range of 0.4–2.5%.^156^

When it comes to propulsive efficiency in the aquatic environment, experimental and theoretical evidence suggests that compliant bioinspired systems may yield better performances than standard propeller-driven robots,^157,158^ and biological studies show that soft organisms indeed benefit from an unprecedented degree of efficiency.^159^ Energy recovery techniques and energy harvesting techniques have been developed for fluidic soft robots to reduce the power consumption, which makes the robot more power efficient.^160–162^

### Sensors

One of the challenges that soft robots face is the balance between softness and load bearing capacity of the robot, where the soft robot needs to be able to withstand its own weight.^149^ The size and weight of the soft robot, or parts of the robot, are two important factors that need to be carefully studied in the design stage.^163^

The discussion about data collection by soft robots naturally leads to soft sensors, that is, sensors that adapt to the change in shape, tension, and extensibility of the body of the robot.^16^ Soft sensors are beyond the scope of this study, but it is worth noticing that recent developments in sensory skins, including material advance (e.g., hydrogel employment),^16^ sensing technique, manufacturing progress, and communication,^164^ are promising also for marine applications.^116,117,165–168^ Moreover aquatic soft sensing can benefit from the development of biomedical soft sensing, as they share similar challenges, such as adhesion, resilience to environmental changes, adaptability, biocompatibility,^167^ and reliability.

Recent studies discuss the complexity of integrating biocompatible materials, memories, communication, and energy harvesting modules, in a unique fully functional platform.^167^

State-of-the-art soft sensors focus on very few sensing modalities, such as temperature and pressure. The soft sensors needed to perform exploration and to generate a sensing based reaction need to be able to embed several sensing modalities,^168^ as neuromimetic architectures suggest.^169^ This requirement poses the new challenge of recording, processing, and generating a response using a minimal amount of time and energy. To this purpose machine learning is a flexible tool to extract and organize information from a vast amount of data.^168^ In particular, Shih *et al.* consider reinforcement learning as a strong tool to develop close-loop control.^168^

### Memory

Another challenging aspect for soft robots is having soft onboard memory. Soft robots are still usually interfaced with hard electronic components that control and power the robot (e.g., batteries and microprocessors). However, soft memory, as soft sensors, would allow the employment of environmental friendly materials reducing the e-waste introduced in the ocean.

Exploiting the digital fluidic logic principle for the onboard memory would reduce the problem of energy support for recording data and reduce the fire hazard constituted by electronic devices around offshore assets, as suggested by recent trends in soft robotics.^144,170–173^ Developing memory using these fluidic logic gates can be quite complex and bulky.^173^ A fluidic S-R latch^144^ is the closest example to a soft memory device. A single S-R latch also requires multiple components (three logic gates and a monostable membrane).^144,173^

Nemitz *et al.*^173^ developed a soft nonvolatile memory device with a bistable membrane, which enables permanent storage of binary information in soft materials. This soft memory device allows writing of information to the memory, as well as erasing the stored information.^173^

According to Calais *et al.*,^174^ chalcogenides is a potential source for providing soft robots with onboard memory capabilities. Chalcogenides, which are natural semiconductors, are also referred to as phase-change materials and are continuing to attract major attention for nonvolatile memory devices with high switching speeds and cycle endurance.^174^

Chalcogenides are good candidates for nonvolatile memory devices because of their phase-changing properties, where they can change from amorphous to polycrystalline structures through thermal annealing.^174^ This phase change significantly increases their electrical conductivity and results in an optical change, allowing them to be used as nonvolatile optical memory materials.^175^ Das Gupta *et al.*^176^ and Li *et al.*^177^ demonstrated the integration of chalcogenides on soft substrates—polydimethylsiloxane (PDMS), where this integration shows the potential of using such materials for onboard memory for soft robotic systems.

### Polymeric pollution

The body of soft robots is often made of polymeric materials. Given the increasing concerns about the accumulation of plastic materials in marine and freshwater environments^178,179^ and especially in light of the toxicity and persistence of many petroleum-based polymers,^180^ it is paramount that the massive deployment of man-made robots in the aquatic environment does not further exacerbate the widespread issue of plastic pollution.

Every year between 4.8 and 12.7 million tonnes of plastic waste ends up in the world's oceans,^181^ with plastic pollution being reported virtually everywhere, from abyssal plains^182^ to polar regions.^183^

Petroleum-based plastics are ubiquitous. They constitute a low-cost, versatile, resilient^184,185^ manufacturing material. But they have a very low biodegradability and persist in the environment for hundreds of years.^186,187^ Therefore, natural and biodegradable materials should be always preferred over synthetic polymers, marking a compromise between environmental impact and technical performance.

Eco-friendly polymers are emerging as an alternative solution to the most common “traditional polymers.”^89–91,188^ Bio-based materials (i.e., produced from renewable resources), however, cannot always be classified as biodegradable.^97,189^ Several products marketed as compostable or biodegradable do not always achieve significant degradation rates when released into the environment.^190^

Ceseracciu *et al.*^90^ estimate their patented starch-based polymer to degrade in Mediterranean waters in 3–6 weeks, but other than that very little is known about the actual degradation times of both traditional and bio-based polymers in the natural environment. Most information originates from laboratory tests^187^; however, the bacterial and physicochemical conditions in natural environments can be drastically different from those achieved in industrial composting plants. Therefore, actual degradation rates of oxo-degradable and compostable polymers are often much slower than expected.^189^

Ideally, selected polymers should meet international standards for biodegradability in the marine environment (e.g., ASTM).^191^ An example is the recent design and large-scale deployment of biodegradable oceanic drifters by the CARTHE Consortium.^192^ After careful considerations, polyhydroxyalkanoates (PHA)—a nontoxic bio-based thermoplastic—were chosen to build the drifter body by industrial injection molding, guaranteeing structural resistance in the marine environment for the duration of the experiment and full bacterial degradation of the drifter body after 5 years at sea with a rate of 0.1 mm/month.^192^

Preferably, all accessories and electronic components need to be nontoxic, favoring the use of lithium batteries, which do not contain lead, mercury, or other hazardous substances. The use of metal should be encouraged, so that it will eventually oxidize in the ocean, as well as other less harmful components such as wood, plant-based materials, or natural rubber. All components should be compliant with the most stringent European and U.S. EPA regulations on hazardous substances, restricting as much as possible the use (and leaching) of toxic compounds such as phthalates, PCBs, PBDs, heavy metals, PAHs, and so on.

Besides the most common thermoplastics such as PVC, PET, PS, and PC, other polymers commonly used in the production of body and skin of aquatic soft robot prototypes are synthetic foams, such as Lycra, silicon rubber, elastomers, latex, acrylic, PDMS, and epoxy resins. Nontoxic bioplastics, manufactured from industrial food waste, are being tested as artificial robotic skins and for the development of biodegradable electronic circuits,^93^ to make the entire device biodegradable.^92,188^

Among innovative materials being tested for the construction of soft robots, there are fluidic elastomers, ionic polymer–metal composites, and piezoceramic materials, whose environmental impacts and biodegradation times are currently unknown.

Hence, to minimize the mass of potentially harmful waste added to the ocean during experiments, it is crucial to always adequately address environmental concerns in the design phase, as well as in the production of soft robots (and their components) designed for release in the natural environment. In addition, the release of innovative polymers and materials, which have never been tested for environmental safety should be always made with caution, and potential negative effects should be ideally tested in laboratory exposure studies or risk assessment procedures before deployment.

## Conclusion and Future Perspectives

This perspective illustrates the application gaps (Fig. 1) and environmental knowledge as driving factors for the future development of marine soft robots.

Offshore industry (Fig. 2) and ocean exploration (Figs. 3 and 4) are due to grow in the oncoming future. Consequently, the demand for autonomous operations in these contexts is deemed to expand. However, long-distance traveling close to the sea bottom and accurate maneuvering close to the sea surface remain two challenging tasks, which currently commercial vehicles are not designed for.

The peculiar features of soft robots, arisen with recent advancement in robotics (Fig. 5), could tackle these contexts of operation. On one hand, compliant bioinspired design promises to enable soft vehicles to achieve higher propulsive efficiency, making them able to navigate over long distances at close proximity with the seabed. On the other hand, nature-inspired propulsive strategy will provide unprecedented maneuvering skills, which, coupled with soft adhesion systems, will enable operation in highly perturbed superficial environments where most of the industrial offshore activities are concentrated.

Flexible sensors will transform these vehicles in nodes of a self-propelling sensor network, and the use of biodegradable materials will make them entirely disposable, minimizing their impact on the environment.

Bioinspiration led the state of the art of soft robots between laboratory based and *in situ* tests, hardly close to full operability. Based on the literature reviewed for this perspective piece, eight core aspects can bring soft robots to full operability: autonomy, communication, efficiency, bio-inspiration, maneuverability and control, memory, resilience, sensors. The development of new sensors, particularly soft sensors, and new biodegradable materials is prime both for deep-sea exploration and for industrial applications. In addition, abyssal expeditions would benefit especially from a design capable to optimize efficiency, communication, memory, and autonomy. Similarly, designs that focus on resilience, maneuverability, and control would strengthen surface operations, important on offshore assets situated in harsh areas.

**FIG. 6.**
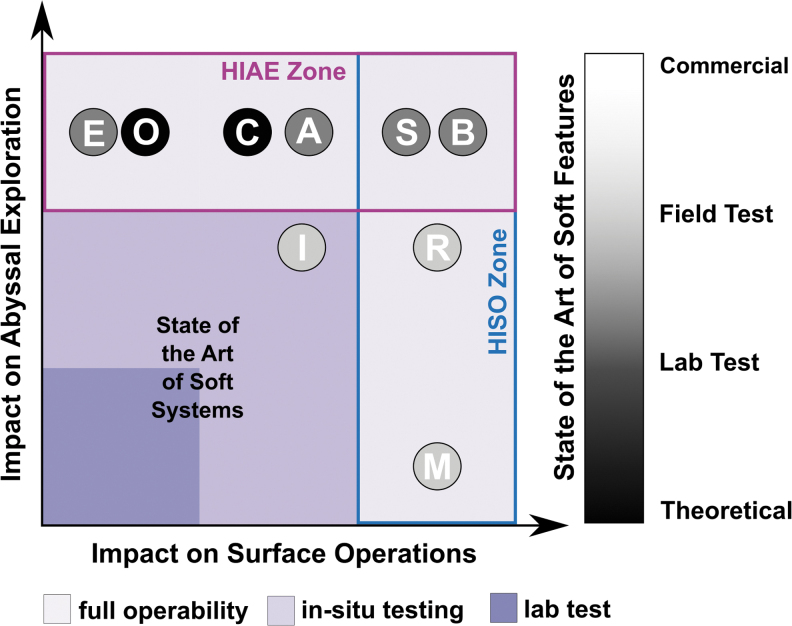
The figure shows the features that are under development and that may have a big impact on aquatic soft robots. The *letters* represent the following: A = autonomy,^57,60,61^ B = biodegradability,^90,92,187^ C = communication,^164^ E = efficiency,^62,149,157^ I = bio-inspiration,^71,111^ M = maneuverability and control,^38,43,72^ O = memory, R = resilience,^183,184^ S = sensors.^117,165,166^ The *letters* at the *top right corner* indicate the areas of development that have a high impact both on abyssal applications and on superficial perturbed water applications. The features that need to be developed the most to move from the prototype stage, where bioinspiration led us, to full operability are enclosed in the HIAE Zone and on the HISO Zone. The *shaded zones* indicate the experimental, *in situ* testing, full operability stages of the aquatic soft robots. The current state of the art of soft systems lays at the intersection between the experimental phase and the *in situ* testing. The *color bar* on the right indicates the key for the individual feature shades, from theoretical to commercial. The assignment of each feature to a specific state of development reflects the result of the present study and wishes to offer a debate space for the community. HIAE, High Impact on Abyssal Exploration; HISO, High Impact on Surface Operation. Color images are available online.

Figure 6 condenses our perspective about the future development of soft robots.

The assignment of each feature to a specific state of development reflects the result of the present study and wishes to offer a debate space for the community.

Soft robots can embrace the challenges embedded in high seas operation and create a new robotic generation capable of monitoring the remotest areas in our planet. We have to be mindful about the design of aquatic soft robots to protect the environment and to contribute to a sustainable development of offshore human activities. Development of these systems will lead to a reduction of manufacturing costs and pave the way to sustainable large-scale deployment of soft robots for monitoring the ocean, leading to an increased spatial resolution for environmental data and remote autonomous asset management.

We wish to encourage present soft robotic studies to develop systems still at a prototype level toward a real world application. Data, even if sparse, from remote environments are extremely precious. In the future soft robotic systems should aim to reach the autonomy, memory, precision, and efficiency of marine rigid robots currently in use. Then their dexterity and biodegradability will be an invaluable added feature in sharp contrast with traditional robots.
